# Insights into the prey of *Vespa mandarinia* (Hymenoptera: Vespidae) in Washington state, obtained from metabarcoding of larval feces

**DOI:** 10.3389/finsc.2023.1134781

**Published:** 2023-02-17

**Authors:** Telissa Wilson, Chris Looney, Luke R. Tembrock, Sapphitah Dickerson, Jessica Orr, Todd M. Gilligan, Mark Wildung

**Affiliations:** ^1^ Washington State Department of Agriculture, Olympia, WA, United States; ^2^ Department of Agricultural Biology, Colorado State University, Fort Collins, CO, United States; ^3^ United States Department of Agriculture, Fort Collins, CO, United States; ^4^ Genomics Core Lab, Washington State University, Pullman, WA, United States

**Keywords:** DNA metabarcoding, invasive species, environmental DNA, diet analysis, fecal pellet, *Apis mellifera*, *Vespa mandarinia*, giant hornet

## Abstract

The northern giant hornet, *Vespa mandarinia* (Hymenoptera: Vespidae), was detected for the first time in North America in 2019. Four nests have since been located and removed in northwestern Washington State as part of an extensive survey and eradication program. This recent introduction into North America has prompted new research on the biology and ecology of *V. mandarinia* to help inform management strategies. In its native range, *V. mandarinia* is known to prey on a variety of insects including the economically important honey bee species *Apis cerana* and *Apis mellifera*. Although *A. cerana* has developed defense mechanisms against attack by *V. mandarinia*, *A. mellifera* have no such defenses and an entire hive can be quickly destroyed by only a few hornets. In North America the hornet has been observed foraging on paper wasps (*Polistes dominula*) and honey bees, but little else is known about prey use in its novel range. To address this knowledge gap, we employed a DNA metabarcoding approach to characterize species detected in larval feces collected from 3 of the 4 Washington *V. mandarinia* nests found to date. Sequences were recovered for 56 species across fourteen orders, of which 36 species were likely prey items and 20 were suspected inquilines. The most frequently detected species were other social Hymenoptera, with *Dolichovespula maculata*, *P. dominula*, and *A. mellifera* present in most samples. All of the species detected, except for *A. mellifera*, represent new prey records for *V. mandarinia*, with eight families of insects newly associated with giant hornets. These results suggest that *V. mandarinia* in Washington preys on an assortment of insects similar to those documented in its native range, and that this new invader has readily incorporated novel species into its foraging and diet.

## Introduction

Vespidae (Hymenoptera) comprises a family of mostly predaceous wasps, including numerous social species. Several vespids in the subfamilies Polistinae and Vespinae – all of which are highly effective, eusocial predators - have been introduced into ecosystems across the globe. Exotic species of paper wasps in the genus *Polistes* and yellowjackets in the genus *Vespula* have spread across Australasia, Polynesia, and North and South America (1). In most cases they have been associated with negative impacts on human health, apiculture, and the environment. Negative impacts stem from competition for resources with native species, predation on endangered and economically important species, reduction in native pollinator populations, and the potential spread of parasites and pathogens (1–4).

More recently, two species of *Vespa* have become established in new habitats where they have caused significant impacts. Multiple nests of *Vespa tropica* were discovered on the island of Guam in 2016 (5). This species has since spread across the island, resulting in measurable damage to apiaries and increased sting incidents (see Rosario et al., this volume). *Vespa velutina*, native to southeastern Asia, is thought to have been introduced to South Korea in 2003 (6) and to France in 2004 (7–9). In South Korea, the species is now found in much of the peninsula, spreading at a rate of up to 20 km/year (3), and it has now been recorded in parts of Japan (10). In Europe, *V. velutina* has expanded from its initial introduction in France to ten other countries (11–13), in some cases spreading at a rate of over 78 km/year (14, 15). In all countries where it has been introduced, *V. velutina* has been associated with increased calls to emergency services and increased loss of honey bee colonies (3, 16). The annual costs of control are predicted to range from 8–11 million Euros annually in Europe (17).

*Vespa mandarinia* (Hymenoptera: Vespidae) is one of the largest hornet species in the world. It is a robust and efficient apex predator of other social Hymenoptera, and coordinated mass attacks late in the season can quickly destroy colonies of honey bees or social wasps [as does the similarly sized and closely related *V. soror*; (18)]. *Vespa mandarinia* was first detected in North America in 2019 (19), and as of December 2022, five nests have been located and destroyed—one in British Columbia, Canada, and four in WA State [(20); Looney et al. in review].

Across its native range, *V. mandarinia* is a regular threat to apiaries and a pest of concern to humans and livestock. The Asiatic honey bee, *Apis cerana*, has evolved defense mechanisms in response to *V. mandarinia* predation which include coordinated attacks on foraging hornets and behaviors to obscure hornet marking pheromones (21–24). The European honey bee, *Apis mellifera*, has not coevolved with *V. mandarinia* and thus is poorly adapted to defend against giant hornet predation. The detections of *V. mandarinia* in North America are concerning due to its potential impact on managed bees and its predicted ability to establish. Several modelling studies indicate the hornet could spread across large swaths of North America if eradication efforts are not successful (25–28).

**Figure 1:**
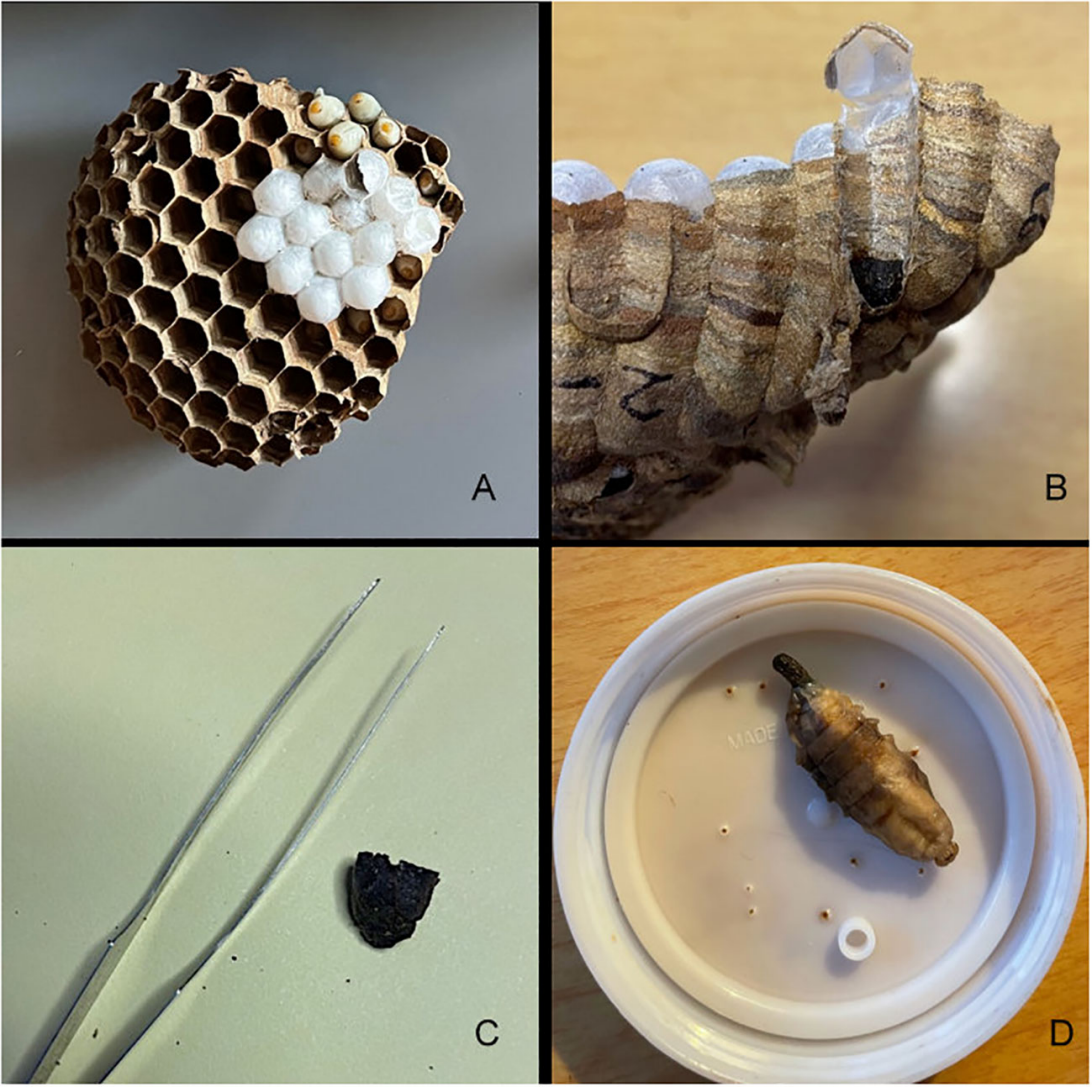
An example of *Vespa mandarinia* nest material and larvae collected in Washington State and used in this study to assess prey items. Clockwise from top left: In **(A)** a comb with capped and uncapped cells is shown, in **(B)** a fecal pellet located in the bottom of cell is indicated by the white arrow, **(C)** is an individual fecal pellet with forceps for scale, and **(D)** is a fecal pellet emerging from a larvae.

In Japan, *V. mandarinia* is known to prey mainly on other social Hymenoptera and opportunistically on a range of other insect taxa, and their diet even extends to scavenging on carrion or other non-insect protein sources. Prey items are collected by the foraging hornets and shaped into “food pellets,” often comprising the thorax of an insect after appendages have been removed. These food pellets are then brought back to the nest and fed to larvae being reared in cells in combs, the number and arrangement of which are species-specific. Larvae defecate in these cells as they develop, and the feces collects in a hard mass at the bottom of the cell (**Figure 1**). Development of larva to adult takes about 34 days (29), and cells are sometimes reused after emergence.

Detailed knowledge of *V. mandarinia* foraging preferences is based primarily on observations from south-central Japan (29, 30). These studies of *V. mandarinia* foraging conducted between 1957 and 1990 identified 19 unique prey species across 5 arthropod orders (Arachnida, Coleoptera, Hymenoptera, Lepidoptera, and Mantodea). Species from Apidae and Vespidae comprised the majority of observed predation events (>78%). These data were based upon visual observations made during foraging or by collecting food pellets from foraging hornet workers, both of which are techniques that require a significant investment in time and which may underestimate the diet breadth. Molecular approaches provide another tool for characterizing prey from insect feces. Multiple recent studies have employed metabarcode sequencing of wasp feces to characterize the diets of different wasp species (e.g., 31–33). Metabarcoding has the advantage of identifying prey items over an entire season of wasp activity and from many individuals in the same nest. In this study, we employed metabarcoding to provide a first glimpse into the foraging preferences of *V. mandarinia* in its newly invaded habitat. These results will serve as a baseline for determining the potential impacts of *V. mandarinia* on native and naturalized species in North America.

## Materials and methods

### Sample collection

Four *V. mandarinia* nests were located and eradicated in WA State between 2020 and 2021, three of which were used in this study. In each case, fecal pellets were collected from the individual cells in each nest, and from empty, occupied by larvae, or capped cells containing pupae/prepupae. For two nests (N1, N2) pellets were removed with forceps or a probe, placed in 1.5 mL tubes, and stored at -20°C until analysis. For the remaining nest (N4; nest 3 was not sampled) most samples were collected into 95% EtOH prior to freezing. Instruments were flame-sterilized and bleached between each sample extraction. Up to 100 mg of feces were collected from each cell for analysis. Sampling effort across nests was uneven based on resources and field work timing. A total of 403 fecal samples were collected (**Table 1**). For N1 and N2, samples were composites of multiple cells and grouped by comb, whereas samples of N4 were kept separate by cell. For N4, gut contents were also collected from late-instar larvae for analysis by dissecting the larva with a scalpel and removing material with micro forceps.

**Table 1:** Sample collection by nest.

Nest	Collection date	Combs sampled	Cells sampled
Nest 1	10/24/2020	5	22
Nest 2	8/25/2021	6	21
Nest 4	9/23/2021	5	360

### DNA extraction, amplification, and sequencing

Sampled fecal material was weighed and 20-40 mg was used for each extraction. A total of 481 fecal samples (including replicate samples) were extracted using a DNeasy Blood & Tissue kit (Qiagen Inc., Hilden, Germany) following the manufacturer’s instructions. Samples were incubated in lysis buffer at 56°C for a minimum of 4 hours prior to subsequent steps. Replicate extractions were performed on samples collected from N1 and N2, and one comb from N4. The remaining samples of all N4 combs were extracted singly, with the exception of 3 samples which included 8 extraction replicates each. DNA extraction purity was assessed using the Nanodrop Lite Spectrophotometer (Thermo Scientific, Wilmington, DE, USA), and DNA was quantified using the Qubit 4 fluorometer with Invitrogen 1X dsDNA HS Assay kit (Invitrogen, Waltham, MA, USA).

A subset of fecal extractions were spiked at the first step of DNA extraction with a synthetic gBlock™ Gene Fragment (IDT, Coralville, IA, USA) designed using the COI sequence from the dodo bird (*Raphus cucullatus*, Accession KX902236) with nucleotide modifications at primer binding sites to match primers used in this study. A CO1 barcode from extinct dodo birds was selected as an internal standard because it provides a biologically realistic nucleotide composition while also being an unlikely prey item of *V. mandarinia* in North America. Based on preliminary comparisons, a total of 400 copies of the dodo bird controls were selected to be spiked into subsequent extractions.

A two-step PCR amplification was used to generate indexed amplicons for sequencing. Step one PCR amplifications were completed in 25 µl reactions and contained 5 µl of molecular grade water, 12.5 µl of 2X Platinum II Hot-start Green PCR Master Mix (Invitrogen), 1.25 µl of MgCl_2_ (50 mM), 0.5 µl of F and R primers (at 10 µM), and 2 µl of DNA template. Desalted oligos containing a 5’ block (5AmMC6) were used to generate the first round PCR product. The primer pair LCO1490 and HCO2198 (34) with M13 tails amplified a 658 bp region of the COI gene with rapid cycling conditions as follows: initial denaturation at 95°C for 1 min., 35 cycles of 2 seconds denaturation at 96°C, anneal at 50°C for 5 seconds, and extension at 72°C for 20 seconds. Final extension was at 72°C for 2 minutes.

Second round PCR products were prepared following the PacBio procedure for preparing multiplexed amplicon libraries using PacBio barcoded M13 primers and SMRTbell^®^ Express Template Prep kit 2.0 (Pacific Biosciences of California, Inc., Menlo Park, CA, USA). Step two PCR amplifications were completed in 25 uL reactions and contained 7.5 µl of molecular grade water, 12.5 µl of 2X Phusion High-Fidelity PCR Master Mix with HF Buffer, 2 µl each of PacBio’s barcoded M13 Forward and Reverse Primer (3 µM), and 1.0 µl of round 1 PCR product. Step two PCR amplification conditions were as follows: initial denaturation at 95°C for 30 seconds, 2 cycles of denaturation at 98°C for 20 seconds, annealing at 60°C for 15 seconds, and extension at 72°C for 60 seconds. Then 20 cycles of denaturation at 98°C for 20 seconds, annealing at 65°C for 15 seconds, and extension at 72°C for 60 seconds. Final extension was at 72°C for 5 minutes. Second round PCR products were quantified with a Qubit 1X dsDNA HS Assay.

Final library construction was preformed using SMRTbell Express Template Prep Kit 2.0 per manufacturer’s guidelines. The normalized second round PCR products were pooled in equimolar amounts for the required total mass of 250-500 ng for library construction. In short, the indexed, pooled amplicons were ligated to SMRTbell adapters and purified using AmPureXP beads (Beckman-Coulter, Brea, CA, USA). Final library concentrations were quality checked using gel electrophoresis and the Qubit flourometer. Final libraries were shipped overnight to the Washington State University Genomics Core Lab in Pullman, WA for sequencing.

Samples were sequenced in a PacBio Sequel II system. Advantages to using the PacBio Sequel platform with SMRTBell Circular Consensus Sequencing (CCS) technology include high output, reasonably improved error rates, and long read sequences which span the entire COI “barcode” region of ~658 bp. A majority of other dietary metabarcoding projects have utilized the shorter read, high-throughput Illumina platforms, but since the introduction of the SMRTbell technology, PacBio has reduced error rates and as a result has seen an increase in applied metabarcoding studies (35–37). SMRTbell libraries were quantified, bound to polymerase using Sequel Binding Kit v3.0, loaded onto a 1M v3 SMRT cell at 16pM and sequenced using V3 reagents for 10 hours.

### Bioinformatics

Circular consensus reads were generated for 500–900 bp fragments with a minimum quality of 0.9999. These high-quality reads were demultiplexed using a minimum index primer quality of QV40. Index separated CCS reads were oriented and trimmed using CLCBio version 10 (Qiagen). Resulting pools of reads for each unique index were clustered to 0.97 ID and centroids and their counts were output using USEARCH v11.0.667 (38). The resulting clusters correspond to Operational Taxonomic Units.

### Error rate and read recovery estimations

To estimate and categorize error produced during PCR, library prep, and/or sequencing the dodo bird gBlock reads that passed QC were compared to the original synthesized sequence. Comparisons were done using custom BLASTn (39) batch searches where the database for comparison included only the synthesized dodo bird gBlock sequence. Three error types were identified: single nucleotide gaps (SNGs), single nucleotide variants (SNVs), and chimeric reads. The SNG errors where totaled from gaps placed in both the query and subject sequences. Chimeras here are defined as a cluster of SNVs (also in some cases SNGs but at a much lower rate) at either the 5’ or 3’ end of a read that originated from another sequence present in the pool as confirmed by searches to the NCBI nr/nt database and comparison to outputs from the pool of origin. Chimeras were tabulated as present or absent (1, 0) in each read cluster. Each error type was calculated per cluster of reads per index set and averaged across a flow cell, resulting in three estimates of error. Read numbers per indexed sample (a single cell worth of feces in most cases) were used to estimate average recovery rate per flow cell given that a known concentration of gBlock was added to each extraction (chimeric reads when present were included in these estimates). Read recovery calculations included samples that did not produce dodo bird reads whereas error calculations did not include these zero values. Average absolute deviation around the arithmetic mean was calculated for each error type for each flow cell. Results from these calculations are given in **Supplemental Table 1**.

### Potential prey analyses

Sequences were assigned to a taxon based on comparisons with the BLAST NCBI (39) and the Barcode of Life (40) databases. All trimmed and clustered reads were batch searched against the NCBI nucleotide database using Geneious Prime (v2.1) with a 0.1 e-value cutoff and default settings for BLASTn searches except for ‘max target sequences’ which was set to 10. Taxonomic identities were only accepted for reads with > 98% similarity to a reference sequence, and resulting reads were compared to BOLD. In most cases, resulting sequence identities were identical between both databases, however the BOLD database was more definitive for Coleoptera and Diptera. Since the goal of this study was to detect potential prey items, we did not include sequences from *V. mandarinia*, *Homo sapiens*, microorganisms, or algae. Data were analyzed as presence-absence of each taxon by sample, nest, and comb, the frequency of occurrence across sample units, and the number of reads of each taxon.

## Results

All indexed samples were successfully amplified and sequenced. After quality filtering and trimming, a total of 748,583 CCS reads were obtained for the three nests. The output of high-quality reads per nest was as follows: 347,625 (N1), 101,217 (N2), and 299,741 (N4). A total of 5,370 clusters were obtained from the high quality reads of all nests. Of these, 47.5% were successfully assigned to an arthropod or allied taxon (this percentage excludes controls, *V. mandarinia*, and various bacteria, fungi, and other microorganisms). Although many studies do not include low frequency reads in their analyses, we chose to evaluate our resulting CCS reads that contained single sequences in the event they represented low abundance prey items. While a majority of the single reads did not add information, a few taxa were identified from only single read sequences. The inclusion of single read sequences was also considered valid based on the results from the internal dodo bird standard, where 25.2% of the recovered sequences were represented by a single reads and in all cases could be identified even when chimeric, albeit below the stringent 98% cutoff in such cases. Error rates inferred from all internal standards were generally low across flow cells with average SNVs per read ranging from 1.1 to 2.5%, and SNGs at 0.04 to 0.1% (**Supplemental Table 1**). Chimeric reads occurred in read clusters from 0.2 to 0.5% of the sequences and in those cases only 14.3% of the time were those chimeric reads the only reads recovered from an extraction. Recovery rates averaged from 2.4 to 4.0 reads per extraction.

**Figure 2:**
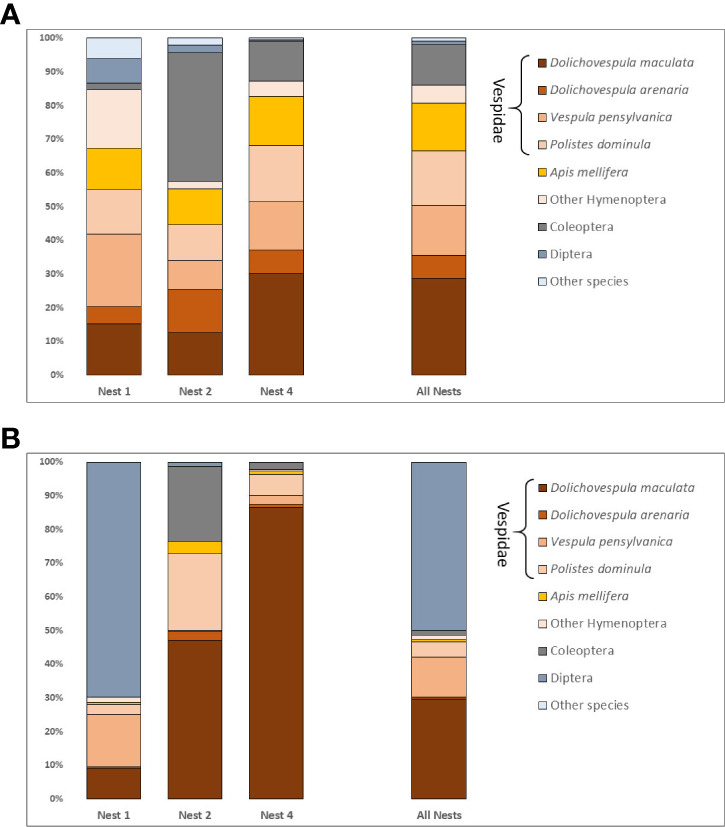
**(A)** Frequency of prey taxa detected for each nest and for all nests combined. Percentages represent a weighted frequency of occurrence based on presence absence counts. **(B)** Relative read abundance expressed as a percentage of all filtered reads for detected prey taxa per nest and for all nests combined.

Fifty-six taxa were identified in the fecal analysis that are conceivably giant hornet food items. They represented eight hexapod orders, one arachnid, two vertebrates, and an earthworm (**Table 2**). The insect taxa identified were distributed across 23 families, the most species rich of which were the Vespidae (10 species) and Cerambycidae (7 species). The number of species varied by nest, with 26 species detected in nest one, 24 in nest two, and 29 in nest four. Social Hymenoptera were the most frequently detected prey items. *Dolichovespula maculata*, the largest yellowjacket in the Pacific Northwest, was detected in 99% of samples. The next most common species were the European paper wasp *Polistes dominula* (64%), the yellow jacket *Vespula pensylvanic*a (57%), and the European honey bee *Apis mellifera* (57%) (**Figure 2A**). Species of Vespidae comprised 70% of the prey items detected by sample. About half of the total species identified were only detected once. Twenty of the species identified are likely to be inquilines, common in decaying trees, or detected because they were otherwise associated with actual prey species, such as *Apocephalus borealis* Brues, 1924, a small phorid parasite of honey bees.

**Table 2:** Species identified from sequencing hornet feces from three *V. mandarinia* nests.

Order	Family	Species	Nest 1	Nest 2	Nest 4
Odonata					
	Aeshnidae	*Aeshna palmata*	X	X	
		*Aeshna* sp.	X		
Blattodea					
	Archotermopsidae	*Zootermopsis* sp.^+^	** **	** **	X
Psocoptera					
	Psocidae	Unknown Genus	X		
Coleoptera					
	Buprestidae	*Agrilus anxius*	X		
		*Buprestis aurulenta*		X	
		*Buprestis laeviventris*		X	
		*Buprestis lyrata*		X	
	Cerambycidae	*Etorofus obliteratus*	X	X	X
		*Etorofus vitiosus*		X	
		*Ortholeptura valida*		X	X
		*Rosalia funebris*		X	X
		*Stictoleptura canadensis*		X	X
		*Xestoleptura crassicornis*		X	
		*Xestoleptura crassipes*			X
	Elateridae	*Ampedus behrensi* ^+^			X
Hymenoptera					
	Formicidae	*Lasius pallitarsus*			X
	Apidae	*Apis mellifera*	X	X	X
	Megachilidae	*Megachile perihirta*			X
	Vespidae	*Ancistrocerus albophaleratus*			X
		*Dolichovespula arenaria*	X	X	X
		*Dolichovespula maculata*	X	X	X
		*Polistes aurifer*	X	X	
		*Polistes dominula*	X	X	X
		*Vespula acadica*			X
		*Vespula alascensis*	X		X
		*Vespula consobrina*			X
		*Vespula germanica*	X		X
		*Vespula pensylvanica*	X	X	X
Lepidoptera					
	Noctuidae	*Noctua pronuba*	X		
	Sphingidae	*Paonias excaecata*	X		
		*Smerinthus cerisyi*		X	
	Nymphalidae	*Vanessa atalanta*	X		
Diptera					
	Dolichopodidae	Unknown Genus			X
	Scatopsidae	*Coboldia fuscipes* ^+^	X		
	Sciaridae	*Cratyna keilini* ^+^		X	
		*Corynoptera cuniculata* ^+^		X	
		*Sciaridae* sp. ^+^	X		
	Syrphide	*Eristalis flavipes*	X		
		*Laphria* sp.		X	X
	Phoridae	Apocephalus borealis*		X	X
		*Triphleba lugubris* ^+^	X		
		*Phoridae* sp. ^+^	X		
	Muscidae	*Muscina prolapsa* ^+^	X		
		*Phaonia tuguriorum* ^+^	X		
	Conopidae	*Physocephala burgessi*			X
	Polleniidae	*Pollenia pediculata* ^+*^	X		
	Sarcophagidaes	*Ravinia querula* ^+^			X
		*Brachicoma devia* ^+^		X	
	Tachinidae	*Strongygaster triangulifera* ^+^	X		
Collembola					
	Entomobryidae	*Willowsia buskii* ^+^		X	
	Tomoceridae	*Tomocerus minor* ^+^			X
Araneae					
	Araneidae	*Araneus diadematus*			X
Crassiclitellata					
	Lumbricidae	*Bimastos rubidus* ^+^	** **	** **	X
Artiodactyla					
	Bovidae	Bos taurus*	X		X
Galliformes					
	Phasianidae	Meleagris gallopavo*		X	X

Several species may be inquilines or associated with the decaying trees in which the nests were located (^+^) or may have been associated with prey species such as with parasitoids or scavenged protein sources (*).

The abundance of reads per taxon (**Figure 2B**) did not always track with the number of times a taxon was detected in a given sample. The most reads were actually recorded for *Triphleba lugubris* (>180,000), a phorid fly that has been associated with vespid nests. This species was only recovered from 12 samples in N1. Excluding *T. lugubris*, there was a generally positive relationship between the frequency of detection per sample and the total number of reads for most other taxa. However, this relationship was only evident when the taxa with the ten next highest reads were considered. 

## Discussion

Field approaches to studying vespid foraging are laborious, and will likely only detect a subset of the prey base. They require fortuitous observations of foraging behavior or focused observations on known locations that likely capture only a subset of foraging activity (18, 41), or collecting food pellets from workers returning to the nest. Each of these techniques is likely to under-sample hornet prey selection, simply due to daily and seasonal limits on research time. Identifying food pellets based on morphology is also challenging, since most diagnostic morphological features are destroyed by the foraging wasps. Because social Hymenoptera remain in the nest during development and feces collect in brood chambers, advances in eDNA analysis offer new techniques which can supplement other approaches to determining prey species, and that can conceivably capture data from the entire active season for the wasps (31).

This study identified 36 taxa that were likely prey items of *V. mandarinia* in northwestern North America. An additional 20 detected species seem more likely to occur as inquilines, even though they could have been eaten by larvae (e.g., Psocoptera). This is probable based on what is known about their biology and their small size. There was one taxon detected that was most likely to have been associated with *V. mandarinia* prey species, but not preyed upon itself. *Apocephalus borealis* was detected in two nests, with only a single read for each. This species of Phoridae is a known parasite of honey bees and bumble bees, and was likely parasitizing honey bees that were preyed upon by *V. mandarinia*.

Relatively few studies of *V. mandarinia* foraging are available, with many concentrating on foraging behavior in apiaries. Mastsuura and Sakagami report *V. mandarinia* predation on 23 taxa in Japan, across five arthropod orders. These observations come from a combination of direct observation and identification of food pellets taken from returning foragers. Our results identified more than twice as many discrete taxa, although some of these (particularly species of Diptera) may not have been directly preyed upon. Our data largely conform to Matsuura’s observations, in that the dominant prey items as measured by the number of reads and the frequency of positive samples were comprised of social Hymenoptera. *Apis mellifera* was well-represented in our results, but was less preyed-upon based on these data than reported by Matsuura’s (although, an earlier version of their table recorded fewer honey bee predation events, suggesting that observations specific to apiaries may have raised these numbers). Aside from the European honey bee, all of the putative prey items were novel. *Vespa mandarinia* seemed to adapt well to preying upon the relatively large yellowjacket *Dolichovespula maculata*. Matsuura and Yamane (1990) report *Vespa simillima* as the second-most frequent observed giant hornet prey item (no other species of Vespa are present in North America). Both *V. simillima* and *D. maculata* create exposed nests, usually in trees, suggesting that *V. mandarinia* is already well-adapted to locating and feeding upon our largest yellowjacket. Interestingly, *Polistes* (mostly just the introduced *P. dominula*) comprised the second-most frequently detected prey species (although only the fourth most as measured by the number of reads), yet in Japan *Polistes* was observed to be less preyed-upon than honey bees, another *Vespa* species, and the yellowjacket *Vespula flaviceps*. The North American data identified species form ten insect families that have not been recorded as *V. mandarinia* prey. The most speciose of these were the beetle families Buprestidae and Cerambycidae. In contrast, the beetle family Scarabaeidae, and the Mantidae (Mantodea), were both recorded in Japan but did not appear in this data. This is likely because of differences in the availability of prey items in each region; mantids are relatively uncommon in northwestern North America, as are large scarabs in the particular area where these nests were located.

Based on our estimates of error from the internal standard and the use of stringent cutoffs for calling a taxon as present the conclusions about prey items presented here are robust. However, such stringency in calls likely lead to some amount of underestimation in diversity of prey items where single chimeric reads would be filtered out. Fortunately, based on our estimates of error, these reads likely occur in very few samples. Future barcoding work of this type might be able to reduce the occurrence of chimeric reads and other PCR associated errors by increasing the extension time in the first round PCR, reducing the cycle number and excluding index sequences with high annealing temperatures (36, 42). Additionally, excluded sequences could be reanalyzed to omit the regions where unrelated sequences are incorporated (e.g., 175 bp from the ends of each sequence). Recovery rate may also have contributed to estimates of diversity where very low abundance prey items might not have been detected; however, at 400 copies per extraction our internal standard is at the lower end of the expected mitochondrial copy number (1,000–5,000 copies) for a single animal cell (43, 44). Improvements to detection of rare prey items could be made by decreasing the number of samples included in a pool, but this would also lower efficiency. In total, the methods presented here provide a flexible and accurate means for studying the prey of *Vespa* and could be applied to the study of microbes or other taxa of interest associated with Vespidae and similar insects.

The data presented here provide more evidence of the adaptability of *V. mandarinia* to novel environments. Habitat suitability models indicate that North America is climatologically suitable for *V. mandarinia* (25, 27, 28), and the discovery of five nests from 2019-2021 confirm this. The data presented here emphasize that *V. mandarinia* will not only find suitable habitat in its introduced range, but will readily adapt to novel prey species, including entire families of insects that have not been previously recorded as prey items. It is important to note, that research on giant hornet predation in its native range has focused on their role as apiary pests, and comes from the work of just a few researchers. It seems likely that future efforts to document giant hornet predation will reveal a more diverse diet even in its native range, whether through metabarcoding approaches or more traditional field observations. The metabarcoding employed here has advantages of generating what seem to be fairly complete prey lists for a relatively small investment of time. A caveat to this method is that other species associated with hornets that are not prey are likely to be detected, and it is hard to verify the provenance of some taxa without direct observation of predation events. As vespids continue to be introduced to new habitats across the globe, dietary metabarcoding can be a helpful tool for estimating the ecological impacts these predators can have upon native insect populations and help to guide management decisions.

## Data availability statement

The data presented in the study are deposited in the NCBI Sequence Read Archive repository, BioProject PRJNA928768; https://www.ncbi.nlm.nih.gov/bioproject/PRJNA928768.

## Author contributions

TW, CL, LT, and TG conceived of and designed the study. TW, SD, and JO prepared and processed samples used in study. TW, CL, LT, and MW performed analyses. TW, CL, LT, and TG contributed to writing of manuscript. All authors contributed to the article and approved the submitted version.

## References

[1] Beggs JR Brockerhoff EG Corley JC Kenis M Masciocchi M Muller F. Ecological effects and management of invasive alien vespidae. Biocontrol (Dordrecht). (2011) 56:505–26. doi: 10.1007/s10526-011-9389-z

[2] Potter-Craven J Kirkpatrick JB McQuillan PB Bell P. The effects of introduced vespid wasps (Vespula germanica and v. vulgaris) on threatened native butterfly (Oreixenica ptunarra) populations in Tasmania. J Insect Conserv (2018) 22:521–32. doi: 10.1007/s10841-018-0081-9

[3] Choi MB Martin SJ Lee JW. Distribution, spread, and impact of the invasive hornet vespa velutina in south Korea. J Asia Pac Entomol (2012) 15:473–7. doi: 10.1016/j.aspen.2011.11.004

[4] Gabín-García LB Bartolomé C Guerra-Tort C Rojas-Nossa SV Llovo J Maside X. Identification of pathogens in the invasive hornet vespa velutina and in native hymenoptera (Apidae, vespidae) from SW-Europe. Sci Rep (2021) 11:11233. doi: 10.1038/s41598-021-90615-7 34045562 PMC8160249

[5] Rosario CA Sablan LR Miller RH Moore A. Greater banded hornet. iNaturalist.org web application . Available at: https://www.inaturalist.org/observations/3663868 (Accessed 27 December 2022).

[6] Kim JK Choi M Ty M. Occurrence of vespa velutina lepeletier from Korea, and a revised key for Korean vespa species (Hymenoptera: Vespidae). Entomological Res (2006) 36:112–5. doi: 10.1111/j.1748-5967.2006.00018.x

[7] Haxaire J Tamisier J-P Bouguet J-P. Vespa velutina lepeletier, 1836, une redoutable nouveauté pour la faune de France (Hym., vespidae). Bull Soc Entomol France (2006) 111:194. doi: 10.3406/bsef.2006.16309

[8] Chauzat MP. Une nouvelle menace pour les abeilles: l’introduction du frelon asiatique vespa velutina en France. Bull Epidémiologique AFSSA. (2009) 32:8–11.

[9] Dillane E Hayden R O’Hanlon A Butler F Harrison S. The first recorded occurrence of the Asian hornet (Vespa velutina) in Ireland, genetic evidence for a continued single invasion across Europe. J Hymenopt Res (2022) 93:131–8. doi: 10.3897/jhr.93.91209

[10] Ueno T. Establishment of the invasive hornet vespa velutina (Hymenoptera: Vespidae) in Japan. Int J Chem Environ Biol Sci (2014) 2:220–2.

[11] Budge GE Hodgetts J Jones EP Ostojá-Starzewski JC Hall J Tomkies V. The invasion, provenance and diversity of vespa velutina lepeletier (Hymenoptera: Vespidae) in great Britain. PLoS One (2017) 12:e0185172. doi: 10.1371/journal.pone.0185172 28950004 PMC5614577

[12] Husemann M Sterr A Mack S Abraham R. The northernmost record of the Asian hornet vespa velutina nigrithorax (Hymenoptera, vespidae). Evol Syst (2020) 4:1–4. doi: 10.3897/evolsyst.4.47358

[13] Lioy S Bergamino C Porporato M. The invasive hornet vespa velutina: distribution, impacts and management options. CABI Rev (2022). doi: 10.1079/cabireviews202217030

[14] Rome Q Muller FJ Touret-Alby A Darrouzet E Perrard A Villemant C. Caste differentiation and seasonal changes in vespa velutina (Hym.: Vespidae) colonies in its introduced range. J Appl Entomology (2015) 139:771–82. doi: 10.1111/jen.12210

[15] Robinet C Suppo C Darrouzet E. Rapid spread of the invasive yellow-legged hornet in France: the role of human-mediated dispersal and the effects of control measures. J Appl Ecol (2017) 54:205–15. doi: 10.1111/1365-2664.12724

[16] Sánchez F Charles X. Notes on the nest architecture and colony composition in winter of the yellow-legged Asian hornet, vespa velutina lepeletier 1836. Vespa velutina Lepeletier 1836 (Hym: Vespidae) its introduced habitat Galicia. (2019) 10:237. doi: 10.3390/insects10080237 PMC6723431 31382493

[17] Barbet-Massin M Salles J-M Courchamp F. The economic cost of control of the invasive yellow-legged Asian hornet. NeoBiota (2020) 55:11–25. doi: 10.3897/neobiota.55.38550

[18] Matsuura M Sakagami SF. Bionomic sketch of the giant hornet, vespa mandarinia, a serious pest for Japanese apiculture. J Ser 6 Zoology. (1973) 19:125–62.

[19] Wilson TM Takahashi J Spichiger S-E Kim I van Westendorp P. First reports of vespa mandarinia (Hymenoptera: Vespidae) in north America represent two separate maternal lineages in Washington state, united states, and British Columbia, Canada. Ann Entomol Soc Am (2020) 113:468–472. doi: 10.1093/aesa/saaa024

[20] Berube C. Giant alien insect invasion averted. Am Bee J (2020) 160:209–14.

[21] Ono M Igarashi T Ohno E Sasaki M. Unusual thermal defence by a honeybee against mass attack by hornets. Nature (1995) 377:334–6. doi: 10.1038/377334a0

[22] Fujiwara A Sasaki M Washitani I. A scientific note on hive entrance smearing in Japanese apis cerana induced by pre-mass attack scouting by the Asian giant hornet vespa mandarinia. Apidologie (Celle). (2016) 47:789–91. doi: 10.1007/s13592-016-0432-z

[23] Fujiwara A Sasaki M Washitani I. First report on the emergency dance of apis cerana japonica, which induces odorous plant material collection in response to vespa mandarinia japonica scouting. Entomological Science. (2018) 21:93–6. doi: 10.1111/ens.12285

[24] Mattila HR Otis GW Nguyen LTP Pham HD Knight OM Phan NT. Honey bees (Apis cerana) use animal feces as a tool to defend colonies against group attack by giant hornets (Vespa soror). PLoS One (2020) 15:e0242668. doi: 10.1371/journal.pone.0242668 33296376 PMC7725375

[25] Alaniz AJ Carvajal MA Vergara PM. Giants are coming? predicting the potential spread and impacts of the giant Asian hornet (Vespa mandarinia, hymenoptera: Vespidae) in the USA. Pest Manage Science. (2021) 77:104–12. doi: 10.1002/ps.6063 32841491

[26] Dai Y Wang H Yang Y. Spatiotemporal distribution analysis of vespa mandarinia based on GM model. J Phys Conf Ser (2021) 1952:42126. doi: 10.1088/1742-6596/1952/4/042126

[27] Nuñez-Penichet C Osorio-Olvera L Gonzalez VH Cobos ME Jiménez L DeRaad DA. Geographic potential of the world’s largest hornet, vespa mandarinia smith (Hymenoptera: Vespidae), worldwide and particularly in north America. PeerJ (2021) 9:e10690. doi: 10.7717/peerj.10690 33520462 PMC7811286

[28] Zhu G Gutierrez Illan J Looney C Crowder DW. Assessing the ecological niche and invasion potential of the Asian giant hornet. Proc Natl Acad Sci U.S.A. (2020) 117:24646–24648. doi: 10.1073/pnas.2011441117 PMC7547231 32963093

[29] Matsuura M. Comparative ecological study of five species of Japan hornets. Tech Bulletin Faculty Agriculture Mie Univ (1984) 69:1–131.

[30] Matsuura M Yamane S. Biology of the vespine wasps. Springer Verlag. (1990) 1–167.

[31] Lefort M-C Beggs JR Glare TR Saunders TE Doyle EJ Boyer S. A molecular approach to study hymenoptera diets using wasp nests. NeoBiota (2020) 63:57–79. doi: 10.3897/neobiota.63.58640

[32] Schmack JM Lear G Astudillo-Garcia C Boyer S Ward DF Beggs JR. DNA Metabarcoding of prey reveals spatial, temporal and diet partitioning of an island ecosystem by four invasive wasps. J Appl Ecol (2021) 58:1199–211. doi: 10.1111/1365-2664.13856

[33] Verdasca MJ Godinho R Rocha RG Portocarrero M Carvalheiro LG Rebelo R. A metabarcoding tool to detect predation of the honeybee apis mellifera and other wild insects by the invasive vespa velutina. J Pest Sci (2022) 95:997–1007. doi: 10.1007/s10340-021-01401-3

[34] Folmer O Black M Hoeh W Lutz R Vrijenhoek R. DNA Primers for amplification of mitochondrial cytochrome c oxidase subunit I from diverse metazoan invertebrates. Mol Mar Biol Biotechnol (1994) 3:294–9. 7881515

[35] Mosher JJ Bowman B Bernberg EL Shevchenko O Kan J Korlach J. Improved performance of the PacBio SMRT technology for 16S rDNA sequencing. J Microbiol Methods (2014) 104:59–60. doi: 10.1016/j.mimet.2014.06.012 24978594

[36] Tedersoo L Tooming-Klunderud A Anslan S. PacBio metabarcoding of fungi and other eukaryotes: errors, biases and perspectives. New Phytol (2018) 217:1370–85. doi: 10.1111/nph.14776 28906012

[37] Wenger AM Peluso P Rowell WJ Chang P-C Hall RJ Concepcion GT. Accurate circular consensus long-read sequencing improves variant detection and assembly of a human genome. Nat Biotechnol (2019) 37:1155–62. doi: 10.1038/s41587-019-0217-9 PMC6776680 31406327

[38] Edgar RC. Search and clustering orders of magnitude faster than BLAST. Bioinformatics (2010) 26:2460–1. doi: 10.1093/bioinformatics/btq461 20709691

[39] Altschul SF Gish W Miller W Myers EW Lipman DJ. Basic local alignment search tool. J Mol Biol (1990) 215:403–10. doi: 10.1016/S0022-2836(05)80360-2 2231712

[40] Ratnasingham S Hebert PDN. Bold: The barcode of life data system (http://www.barcodinglife.org): BARCODING. Mol Ecol Notes (2007) 7:355–64. doi: 10.1111/j.1471-8286.2007.01678.x PMC1890991 18784790

[41] Choi M. Foraging behavior of an invasive alien hornet (Vespa velutina) at apis mellifera hives in Korea: Foraging duration and success rate. Entomological Res (2021) 51:143–8. doi: 10.1111/1748-5967.12510

[42] Ho M Moon D Pires-Alves M Thornton PD McFarlin BL Wilson BA. Recovery of microbial community profile information hidden in chimeric sequence reads. Comput. Struct. Biotechnol. J. (2021) 19:5126–39. doi: 10.1016/j.csbj.2021.08.050 PMC8453192 34589188

[43] Moraes CT. What regulates mitochondrial DNA copy number in animal cells? Trends Genet (2001) 17:199–205. doi: 10.1016/s0168-9525(01)02238-7 11275325

[44] Liu R Jin L Long K Tang Q Ma J Wang X. Analysis of mitochondrial DNA sequence and copy number variation across five high-altitude species and their low-altitude relatives. Mitochondrial DNA B Resour (2018) 3:847–51. doi: 10.1080/23802359.2018.1501285 PMC7799994 33474342

