# Supplementary material for: Insights into the prey of Vespa mandarinia (Hymenoptera: Vespidae) in Washington state, obtained from metabarcoding of larval feces
Source: Front Insect Sci. 2023 Feb 17;3:1134781. doi: 10.3389/finsc.2023.1134781 (PMC10926418; doi:10.3389/finsc.2023.1134781)
Supplement: Supplementary file 1 [file Table_1.docx]

Supplemental table 1. Error rates calculated for different error types per flow cell using an internal standard gBlock with synthesis based on a dodo bird mtDNA CO1 sequence which was added at the first step of DNA extraction at 400 copies. The first two error type calculations are given in average percent error per barcode sequence based on the internal standard across all extractions in a given pool and sequenced on a single flow cell, the average number of nucleotide errors per barcode sequence based on the internal standard across all extractions in a given pool and sequenced on a single flow cell, and the absolute deviation around the arithmetic mean for the average number of nucleotide errors (each value separated by slashes). Chimeric reads are presented as the average number of internal standard reads containing chimerism per extraction across a pool sequenced on a single flow cell. Recovery rate is given in average reads per extraction for the internal standard across the entire pool sequenced on a single flow cell. Final values in each column are absolute deviation around the arithmetic mean associated with the proceeding value. Abbreviations are: SNV; single nucleotide variant and SNG: single nucleotide gap.

| **Flow Cell** | **average SNV error rate** | **average SNG error rate** | **average number of chimeric sequences** | **average recovery rate in reads** |
| --- | --- | --- | --- | --- |
| 1 | 1.10/7.83/12.03 | 0.04/0.31/0.47 | 0.20/0.18 | 2.37/2.61 |
| 2 | 2.44/17.31/24.09 | 0.08/0.57/0.70 | 0.28/0.37 | 2.56/2.40 |
| 3 | 2.48/17.58/24.26 | 0.13/0.94/1.03 | 0.50/0.39 | 4.02/5.76 |
